# Coercive containment measures for the management of self‐cutting versus general disturbed behaviour: Differences in use and attitudes among mental health nursing staff

**DOI:** 10.1111/inm.13006

**Published:** 2022-04-17

**Authors:** Geoffrey L. Dickens, Leah Hosie

**Affiliations:** ^1^ 5995 Mental Health Nursing Northumbria University Newcastle upon Tyne UK; ^2^ Mental Health Nursing Abertay University Dundee UK

**Keywords:** attitudes, mental health nurses, physical restraint, seclusion, self‐harm

## Abstract

Self‐harm is common in mental health facilities, and coercive containment measures are sometimes used to manage it. Nurses' attitudes towards these measures have been investigated in relation to disturbed behaviour in general, but rarely to self‐harm specifically. We therefore investigated mental health nurses' use of and attitudes towards coercive measures (seclusion, restraint, intermittent and constant observations, forced intramuscular medication, and PRN medication) for self‐cutting management compared with for disturbed behaviours in general using a cross‐sectional, repeated measures survey design. Participants were *N = *164 mental health nursing staff. Data collection was via a questionnaire comprising validated attitudinal measures. The study is reported in line with STROBE guidelines. Physical restraint (36.6%), forced intramuscular medication (32.3%) and seclusion (48.2%) had reportedly been used by individuals for self‐cutting management. Respondents disapproved of using each coercive measure for self‐cutting more than they did for disturbed behaviour in general with the exception of PRN medication. Attitudes to coercive measures differed across target behaviours. Hence, nurses who had used each measure for managing self‐cutting disapproved of it less for that purpose than those who had not. Nurses who had used coercive techniques for self‐cutting management had less desirable attitudes to their use. We cannot say whether prior use of these techniques led to increased approval or whether greater approval led to an increased willingness to use them. Reducing the use of coercive techniques for self‐harm will require attitudes that support its use to be challenged. Less coercive techniques should be encouraged. Harm reduction techniques offer one such alternative.

## INTRODUCTION

Coercion is the act or process of compelling a person to act through the use of threats including force (Merriam‐Webster 2021). The history of the use of coercion in mental health services internationally is as old as the services themselves (Molodynski *et al*. 2016). Beyond the involuntary admission and detention of individuals in mental health services, sanctioned coercive practices include physical restraint, seclusion, and forced neuroleptic medication (Raboch *et al*. 2010). Researchers have sometimes identified measures such as constant or intermittent observations, and the administration of *pro re nata* (‘PRN’ or ‘as needed’) neuroleptic medication as coercive (e.g. Bowers *et al*. 2007). The use of coercive measures is prevalent internationally; in a survey of involuntarily detained patients in 10 European countries, 8%, 36%, and 56% had been placed in seclusion, restrained, and been forcibly medicated, respectively (Raboch *et al*. 2010). There has been growing concern about their continued use and momentum has grown for no use and minimal use approaches from service users and professionals alike (Kinner *et al*. 2017; Morandi *et al*. 2021). Further, these ambitions have been enshrined in public policy (Department of Health 2014).

One area that has received research attention is the attitudes of nurses and other mental health professionals to the use of coercive measures (Bowers *et al*. 2007; Dickens & Hosie 2018; Jalil *et al*. 2017; Vandamme *et al*. 2021; Whittington *et al*. 2009). One feature of the empirical literature about mental health nurses' attitudes to coercion is that they are investigated and discussed as a response to disturbed behaviour in general including aggression (e.g. Whittington *et al*. 2009) or to aggression specifically (e.g. Jalil *et al*. 2017). An aspect that has hitherto largely evaded investigation is their use for management of self‐harm specifically.

## Background

Self‐harm has been defined as any form of self‐inflicted injury or poisoning (NICE 2013). It is an established predictor of suicide; others include suicidal intent, poor physical health, and male gender (Chan *et al*. 2016). While suicidality should not be underestimated, it is not always or even usually present in self‐harm; for example, in Chan *et al*.'s systematic review of prospective studies of risk factors for suicide, between 12% and 28% of almost 10 000 participants across three investigations were judged to have suicidal intent. Acts of self‐harm occur for a variety of reasons and the level of lethal intent behind acts includes ‘partial or nonzero’, (Posner *et al*. 2014: p.24), also suggesting that suicidal intent is not always present in these acts. NICE (2004) describe self‐harm as an expression of distress with a diverse range of causes, while Sutton (2007) defines it as a coping strategy utilized in the face of intolerable distress with the aim of restoring emotional balance. A spectrum of nonsuicidal self‐harm behaviour has been proposed (Muehlenkamp 2014) from mild (such as occasional binge‐drinking) to moderate (such as intentionally bruising oneself) to severe (such as self‐cutting). Self‐harm presents a significant ongoing healthcare issue. For example, in England, there was an increase in self‐reported self‐harm across the lifetime from 2.4% to 6.4% between 2000 and 2014 (McManus *et al*. 2019).

Self‐harm in inpatient mental health services is common. A systematic review (James *et al*. 2012a, 2012b) noted a mean of 17.4% of mental health inpatients self‐harming within the ward setting. Nursing reactions to self‐harm vary from proactive and therapeutic to reactive and coercive (Dickens & Hosie 2018). Research on the epidemiology of the use of coercive measures like seclusion and restraint for self‐harm are less prominent in the literature than for aggression and disturbed behaviour in general but there are numerous indications that they are indeed used for this purpose. A number of studies identified self‐harm as an antecedent to restraint in Stewart *et al*.'s (2009) review and, more recently, in a survey of 144 cases of seclusion and restraint in a forensic psychiatric hospital (Kuivalainen *et al*. 2017), 35 (24.3%) were determined to have been instigated due to a patients' self‐directed harmful behaviour alone. Increasing an individual's level of observation to prevent self‐harm is also a favoured response (Stewart *et al*. 2009). The efficacy of observation as a helpful technique has been questioned by Stewart *et al*. (2009) who noted that not only is there no identifiable link between observation and self‐harm reduction but also that a reduction in observation did not lead to a corresponding increase in self‐harm. In fact, observation can act as a catalyst for further so‐called challenging behaviours, resulting in aggression, non‐compliance with medication, refusal to eat, and increased use of PRN medications and physical restraint techniques (Stewart *et al*. 2011).

In terms of the acceptability to service users of the use of coercive interventions for the management of disturbed behaviour in general, Whittington *et al*. (2009) reported that psychiatric inpatients were significantly less supportive of a range of interventions including intermittent and constant observations, PRN, restraint, and seclusion than were inpatient staff on the validated attitudes towards containment measures questionnaire (ACMQ; Bowers *et al*. 2004). We think, however, that results about the use of such measures *in general* cannot and should not be assumed to generalize to their use for the management of self‐harm *in particular*.

Nurses' behavioural and attitudinal reactions to self‐harming behaviours and the interventions they implement to manage them form an important aspect of the inpatient service user experience. Quantitative tools designed to measure these responses include the Attitudes towards Deliberate Self‐Harm Questionnaire (ADSHQ; McAllister *et al*. 2002) and the Self‐Harm Antipathy Scale (SHAS; Patterson *et al*. 2007b). The former is focused on nurses' experience of having the toolkit of skills, knowledge, and characteristics necessary to adequately and appropriately care for this client group, while the latter examines the relationship between the nurse and the service user and the emotional responses that occur as a result. Studies utilizing these tools and others, or exploratory qualitative approaches, have garnered a variety of responses (Karman *et al*. 2014). Of studies conducted in mental health settings, staff responses suggest that there is empathy with and understanding of self‐harm in individuals and self‐efficacy regarding ability to effectively manage self‐harm (Dickinson *et al*. 2009; Wheatley & Austin‐Payne 2009; Wilstrand *et al*. 2007). Concurrently, studies suggest there is also frustration (O'Donovan & Gijbels 2006), irritation, anger, shame and disgust (Thompson *et al*. 2008), and antipathy (Dickinson *et al*. 2009). Education seems to have positive effects on antipathy at least in the short term (Patterson *et al*. 2007a; Wheatley & Austin‐Payne 2009) and mental health nurses seem to have less antipathy than adult nurses (Patterson *et al*. 2007b).

Recently, the Attitudes to Self‐Cutting Management Scale (ASC‐Me; Hosie & Dickens 2018) has been developed to help address the gaps outlined above by measuring attitudes towards management techniques, including coercive techniques, for acts of self‐harm. The scale examines the case of self‐cutting only rather than self‐harm generally because it is the only method of self‐harm that has been suggested as a candidate for a full range of harm reduction approaches including advice on safe cutting, provision of sterile blades and remaining present during an act of cutting. For example. Gutridge (2010) has suggested that service users being allowed the opportunity to utilize their coping strategies in the short term and under controlled circumstances enhances autonomy and ultimately improves the longer term therapeutic relationship. We are unaware of any proposal for the use of such approaches in relation to, for example, self‐poisoning, self‐immolation, or autoasphyxiation. In a previous paper (Hosie & Dickens 2018) the attitudes of mental health nursing staff were compared with those of a sample of ex‐service users who had experience of self‐cutting in the inpatient environment. Nurses were more approving of a wide range of interventions than were patients including some coercive techniques (seclusion, restraint) but not others (forced intramuscular medication, intermittent and close observations), and more approving of some therapeutic activities (psychological interventions, distraction, care planning, wound care advice, provision of a first aid kit). There was no difference between groups regarding more controversial harm reduction techniques (provide sterile razors, remain present during cutting). Further, despite being more approved of by nurses than patients, seclusion, restraint, and forced IM medication all ranked less positively than all other named interventions for both groups with the exception of clearly inappropriate interventions (treatment refusal, provision of inappropriate treatment).

The aim of the current study is to focus specifically on mental health nurses' use of and attitudes toward coercive interventions for the management of self‐cutting behaviour relative to that of the same interventions when used for disturbed behaviour more generally. Given that nurses rate some of these techniques more positively than those who are subject to them, and despite their continued use despite some nurses' apparent disdain, it is anticipated that exploration of attitudes towards coercive interventions based on the ostensible reason for their use would have implications for education and practice including informing educational initiatives to reduce their use. Specific study objectives are to determine:

- the prevalence of mental health staffs' prior use of coercive measures to manage self‐cutting behaviour.
- whether mental health staffs' attitudes towards the use of coercive measures differs depending on the target managerial issue.
- whether mental health staffs' attitudes to self‐cutting management differ depending on their prior experience of using coercive measures.
- differences in attitudes towards coercive self‐cutting management as a function of gender, age, position or length of service.

## METHODS

The study has one previously published output (Hosie & Dickens 2018) in which an attitudes towards self‐cutting management scale was developed and then used to compare similarities and differences between the evaluations of mental health nursing staff and people with lived experience of having their self‐cutting behaviour managed in an inpatient setting. This paper focuses on the reported use of and attitudes towards a subset of six coercive interventions for both the management of self‐cutting and the management of disturbed behaviour more generally among mental health nursing staff. The interventions are physical restraint, seclusion, forced intramuscular medication, intermittent observations, close observations and PRN medication. The paper is reported in line with STROBE guidelines (von Elm *et al*. 2008).

### Design

The study used a cross‐sectional, repeated measures survey design utilizing both online and paper‐based data collection. Mental health staff participants were registered mental nurses, nursing and healthcare assistants, and student mental health nurses. The sample was opportunistic, comprising self‐selecting respondents. Participants were drawn from two NHS Health Boards in Scotland via a paper‐based survey, and from UK‐wide via a web‐based survey (Survey MonkeyTM). A link to the online survey was circulated via fora including the Mental Health Nurse Academics UK (https://mhnauk.wordpress.com/) mailing list. This is a grouping with representatives from every university providing pre‐registration mental health nurse training in the UK. Potential staff participants were eligible if they had recent experience of working in a mental health inpatient ward in which self‐harm by cutting had occurred. Data were collected in the period April to June 2017. The minimum sample size for the original study was determined to be 150–200 based on guidelines for reporting exploratory factor analysis where communalities exceed 0.4 (Worthington & Whittaker 2006). For the current analysis, it was determined (Kane 2022) that a difference between respondents who had and had not previously used seclusion for the management of self‐cutting of *SD* = 1 (large effect size) would be detected with a sample size of *N = *52 (*n = *26 per group; α = 0.05, β = 0.05, 95% power).

The project was approved by the Abertay University Research Ethics Committee and NHS Tayside Research & Development Department. Potential participants were provided with full written details; on this occasion, consent was taken to be implied by return or online completion of the questionnaire. All participants were provided with details of possible sources of support in the event of any distress. Participants were also offered the option of contacting either of the researchers, both experienced mental health nurses, for a debrief.

### Data collection

#### Demographics and experience

Information about participant age, gender, role, and length of service were collected via a custom‐designed questionnaire. Participants were also asked to indicate whether they had previously used each of the six coercive techniques (i) for the management of disturbed behaviour in general and (ii) for the management of self‐cutting in particular.

#### Attitudes to Containment Measures Questionnaire (ACMQ)

The ACMQ is an 11‐item tool which measures attitudes towards commonly used containment measures for managing disturbed behaviour on mental health wards. In the original validation version of the ACMQ (Bowers *et al*. 2004), respondents were asked to rate each method in respect of its effectiveness, acceptability, respectfulness, safety for staff, safety for the patient, and preparedness to use (staff) or preparedness to be subject to (service users). Response for each domain is on a five‐point Likert scale (‘strongly disagree’ to ‘strongly agree’). Higher scores represent greater approval of the technique in terms of that domain. Later versions of the ACMQ, and that used here, have simply asked respondents about ‘acceptability’ since the validation study found high communalities within ACMQ factors – in effect, ratings of acceptability, effectiveness and so on are highly inter‐related for each containment method and it was deemed acceptable to reduce measurement for each factor to a proxy item. We only administered the six items related to coercive measures (physical restraint, forced intramuscular medication, seclusion, intermittent observations, constant observations, and PRN medication).

#### Attitudes to Self‐cutting Management Scale (ASc‐Me)

The ASc‐Me (Hosie & Dickens 2018) was developed by the current authors to measure attitudes towards a full range of 17 approaches to care and management of inpatients who display self‐cutting behaviour. Similarly to the ACMQ, the ASc‐Me comprises a short description of each intervention and then asks respondents to rate it on a series of five‐point Likert scales in respect of its acceptability, effectiveness, respectfulness, safety for staff, safety for the patient, and their preparedness to use it (staff) or their preparedness to be subject to it (service users) for management specifically of self‐cutting. Again, a higher score represents greater approval of the technique. In the validation study, very high communalities were reported for all techniques (*M* Cronbach's α for the six techniques reported here was 0.91). The techniques reported on in this study are close observations, intermittent observations, seclusion, forced IM medication, physical restraint, and voluntary PRN administration.

### Data analysis

Descriptive statistics were used to describe the study sample. Data were examined for normality of distribution (skewness and kurtosis statistics). Prior use of each technique for its stated purpose was compared using Fisher's exact test. To produce a single score for each ASC‐Me technique, and thus to allow direct comparison between ratings of similar interventions for the management of disturbed behaviour and self‐cutting behaviour, we computed the mean of the six ratings. To check they were similar to the single ‘acceptability’ rating for each technique, we calculated Pearson's *r* (*M* = 0.892). Reflecting the study hypotheses, repeated measures t‐tests were conducted to compare respondents' respective evaluations of the use of each technique for management of self‐cutting and of disturbed behaviour in general. Demographic (gender, age, job role, years of experience) differences on attitude ratings were examined using independent samples *t*‐tests and one‐way ANOVAs. Linear regressions were conducted to determine whether prior use of each coercive intervention for self‐cutting management specifically and for management of disturbed behaviour generally plus any other potential covariates identified in the univariate analyses predicted its related attitudinal evaluation.

In reporting our findings, we have chosen to use the ‘desirable’ attitude as the reference point. That is to say, we compare *disapproval* (desirable) of coercive interventions to *approval* (undesirable). Thus, when reading results, please note that the terms ‘more disapproval’ and ‘greater disapproval’ indicate more of the ‘desired’ attitude. While this ascribed status may be less justifiable for, say, the use of voluntary PRN medication than it is for seclusion, it is intended to facilitate consistency in reporting.

## RESULTS

In total, *N = *175 people participated in the survey. The proportion of those accessing the questionnaire who went on to complete it was 65.0%. Of these, *n = *164 completed all relevant items reported here with the remaining *n = *11 not completing all ACMQ items, and we therefore excluded their data from the study. The excluded participants did not differ significantly from included ones on any of the demographic variables or on ASC‐Me coercive interventions ratings. Characteristics of included participants are detailed in Table 1.

**Table 1:** Participant characteristics

	*n* (%)
Age (years)	
18–29	35 (21.3)
30–39	33 (20.1)
40–49	52 (31.7)
50+	44 (26.8)
Gender	
Male	38 (23.2)
Female	125 (76.2)
Missing	1 (0.6)
Length of service (years)	
0–5	52 (31.7)
6–10	12 (7.3)
11–20	36 (22.0)
>20	64 (39.0)
Role	
Registered Nurse	130 (79.3)
Health Care Assistant	7 (4.3)
Student Nurse	27 (16.5)

Table 2 shows that previous use of all six coercive techniques was significantly more prevalent for management of disturbed behaviour in general than for self‐cutting management in particular. For constant and intermittent intervention, and for voluntary PRN, more than half of respondents had used the interventions both for managing self‐cutting and disturbed behaviour in general (range 55.5–77.4%). Forced IM medication, restraint, and seclusion had been used by 28.7%, 33.5%, and 42.1% of all participants for both stated purposes, respectively. There was a moderate degree of correlation between previous use of each coercive technique for management of self‐cutting and of general disturbed behaviour (*M r* = 0.33, range 0.185–0.416).

**Table 2:** Use of coercive containment measures for self‐cutting versus general disturbed behaviour

	Have used for self‐cutting management *n* (%)	Have used for management of disturbed behaviour *n* (%)	*X^2^*	*P*
Physical restraint	57/164 36.6%	115/164 70.1%	28.99	<0.001
Forced IM medication	53/164 32.3%	101/164 61.6%	24.3	<0.001
Seclusion	79/164 48.2%	115/164 70.1%	21.57	<0.001
Close observations	106/164 64.6%	154/164 93.9%	5.59	=.024
Intermittent observations	96/164 58.5%	133/164 81.1%	26.21	<0.001
PRN	133/164 81.1%	152/164 92.7%	13.13	=0.002

Table 3 shows that staff attitudes towards the use of four coercive measures were more disapproving when used for the management of self‐cutting than when used for disturbed behaviour more generally. Effect sizes were large, except in the case of intermittent observation (moderate). The exception was the use of voluntary PRN which respondents rated equally positively for use in both circumstances. While respondents differed significantly in their rating of most techniques based on its ostensible purpose, there was a positive correlation between scores for all techniques: intermittent observations *r* = 0.61 *P *< 0.001; close observations *r* = 0.22 *P* = 0.004; forced IM *r* = 0.44 *P *< 0.001; seclusion *r* = 0.34, *P* < 0.001; PRN *r* = 0.45, *P* < 0.001; physical restraint *d* = 0.32 *P* < 0.001.

**Table 3:** Staff disapproval of the use of management of self‐cutting versus management of disturbed behaviour using similar coercive techniques

	Self‐cutting *M (SD)*	Disturbed behaviour *M (SD)*	*t*	*P*	*d*
Physical restraint	3.56 (0.97)	2.63 (1.12)	9.788	<0.001	0.89
Forced IM medication	3.83 (1.04)	2.76 (1.16)	11.717	<0.001	1.00
Seclusion	2.97 (1.11)	1.30 (0.46)	18.41	<0.001	2.09
Close observations	2.89 (0.86)	2.04 (0.90)	9.88	<0.001	0.97
Intermittent observations	2.76 (0.96)	2.19 (1.03)	8.356	<0.001	0.67
PRN	1.95 (0.71)	1.85 (0.86)	1.140	0.256	0.13

Table 4 shows that for all techniques nurses who had used each technique for the management of self‐cutting behaviour were less disapproving of it for that use compared with those who had not used it for that purpose. Effect sizes were notably large for the most coercive management strategies. Further, staff with greatest length of experience were most disapproving of the use of forced IM medication and physical restraint. There were no significant differences between ratings for any technique by gender. Registered nurses were more disapproving of the use of constant observations (*M*[*SD*] 3.0 [0.84] vs. 2.60 [0.86], *t* = −2.58, *P* = 0.01, *d* = 0.47) and intermittent observations (2.83 [0.97] vs. 2.42 [0.71] *t*=−2.39, *P* = 0.018, *d* = 0.48) than were HCAs and students. There were significant length of service differences on attitudes to forced IM medication (*F* (2, 155) = 4.93, *P* = 0.008), intermittent observations (*F* (2, 155) = 4.13, *P* = 0.018) and physical restraint (*F* (2, 155) = 3.86, *P* = 0.023). Post hoc Tukey tests revealed that there was significantly more disapproval of forced IM medication among nurses in categories of six plus years' experience compared with those with less experience; for intermittent observations, there was significantly more disapproval in those with 10+ years' experience compared with those with less than five years; for physical restraint, the same pattern as for intermittent observations was evident. Similar differences were found on the variable age with older respondents more disapproving of the same interventions. However, given the strong covariance of age and years of experience (*r* = 0.793), only the latter was entered into the linear regression analyses.

**Table 4:** Approval of techniques for self‐cutting management as a function of whether they have previously used techniques for this purpose

Coercive intervention	Has used the technique for self‐cutting management	*n*	ASC‐ME *M(SD)*	*t*	*P*	*d*
Physical restraint	Used	57	2.9 (0.93)	−7.46	<0.001	1.24
	Not used	107	3.92 (0.79)			
Forced IM medication	Used	53	2.99 (0.87)	−8.52	<0.001	1.37
	Not used	111	4.23 (0.86)			
Seclusion	Used	79	2.38 (0.88)	−8.13	<0.001	1.24
	Not used	85	3.51 (1.02)			
Close observations	Used	106	2.76 (0.87)	−2.94	=0.004	0.47
	Not used	58	3.14 (0.81)			
Intermittent observations	Used	96	2.52 (0.92)	−4.16	<0.001	0.63
	Not used	68	3.07 (0.90)			
PRN	Used	133	1.85 (0.61)	−3.81	<0.001	0.70
	Not used	31	2.36 (0.9)			

Table 5 shows that previous use of each technique specifically for self‐cutting management contributed significantly to the prediction of related attitudes in all six cases; further, in all cases, previous use of the technique for that purpose predicted a less disapproval of the use of the technique for management of self‐cutting. Previous use of each technique for management of disturbed behaviour in general only contributed to attitudes towards use for self‐cutting management in two cases: physical restraint and seclusion. In both of these cases, the contribution was associated with more disapproval of use of these techniques for self‐cutting management.

**Table 5:** Linear regression analyses of potential predictors of attitudes to each coercive intervention

Intervention	Predictor	*B* (95% CI)	*P*
Intermittent observations	(Constant)	2.15 (1.51, 2.79)	<0.001
	5+ years length of service (vs. <5 years)	0.27 (0.09, 0.44)	0.003
	HCA/Student (vs. Nurse)	0.23 (−0.15, 0.62)	0.24
	Previous use for self‐cutting (vs. no)	−0.73 (−10.05, −0.42)	<0.001
	Previous use for disturbed behaviour (vs. no)	−0.04 (−0.42, 0.35)	0.853
	Adjusted *R* ^2^ = 0.184 (*F* (4, 145) = 9.39), *P *< 0.001		
Constant observations	(Constant)	2.14 (1.4, 2.89)	<0.001
	HCA/Student (vs. Nurse)	0.53 (0.22, 0.85)	0.001
	Previous use for self‐cutting (vs. no)	−0.44 (−0.72, −0.17)	0.002
	Previous use for disturbed behaviour (vs. no)	0.09 (−46, 0.63)	0.76
	Adjusted *R* ^2^ = 0.107 (*F* (3,160) = 6.39), *P *< 0.001		
Forced IM	(Constant)	3.61 (3.25, 3.97)	<0.001
	5+ years length of service (vs. <5 years)	0.24 (0.09, 0.40)	0.003
	Previous use for self‐cutting (vs. no)	−1.29 (−1.61, −0.97)	<0.001
	Previous use for disturbed behaviour (vs. no)	0.06 (−0.27, 0.39)	0.732
	Adjusted *R* ^2^ = 0.352 (*F* (3, 145) = 27.84), *P *< 0.001		
Physical restraint	(Constant)	3.14 (2.80, 3.48)	0.001
	5+ years length of service (vs. <5 years)	0.25 (0.1, 0.4)	0.001
	Previous use for self‐cutting (vs. no)	−1.33 (−1.63, −1.03)	<0.001
	Previous use for disturbed behaviour (vs. no)	0.43 (0.1, 0.76)	0.011
	Adjusted *R* ^2^ = 0.369 (*F* (3, 145) = 28.26), *P *< 0.001		
PRN	(Constant)	2.38 (1.97, 2.78)	<0.001
	Previous use for self‐cutting (vs. no)	−0.5 (−0.78, −0.22)	0.001
	Previous use for disturbed behaviour (vs. no)	0.03 (−0.45, 0.40)	0.900
	Adjusted *R* ^2^ = 0.067 (*F* (2, 161) = 6.94), *P *= 0.001		
Seclusion	(Constant)	3.45 (3.24, 3.66)	<0.001
	Previous use for self‐cutting (vs. no)	−1.17 (−1.47, −0.88)	<0.001
	Previous use for disturbed behaviour (vs. no)	0.4 (0.03, 0.77)	0.034
	Adjusted *R* ^2^ = 0.275 (*F* (2, 161) = 31.92), *P *< 0.001		

N.B. Positive B coefficient indicates an increase in disapproval negative B coefficients indicate a decrease in disapproval.

## DISCUSSION

Self‐harm presents a serious challenge to providers of inpatient mental health services. Its occurrence precedes a sizeable proportion of the use of coercive techniques in these settings (Kuivalainen *et al*. 2017; Stewart *et al*. 2009). There is little evidence that those techniques are effective in reducing self‐harm and they may actually be counterproductive. In the current study, we found that participants' reported use of coercive techniques for disturbed behaviour in general was more prevalent than for self‐cutting. While this is not surprising per se, a sizeable proportion of respondents reported having used notably coercive measures for self‐cutting management; one in three respondents had used seclusion and physical restraint for this purpose. There were statistically significant correlations whereby respondents who reported previous use of each technique for management of disturbed behaviour in general also reported its use for self‐cutting specifically. However, they were moderate in size suggesting there is no inevitable link between use for one and the other and that there is scope to separate out self‐harm management for attention: for example, education and campaigning could deliver a ‘minimise restraint for self‐harm’ message. While broad‐brush approaches aimed at reducing coercive interventions such as Safewards (Bowers *et al*. 2015) have had demonstrable success, it is largely focused on reducing aggression and violence. Similarly, REsTRAIN YOURSELF (Duxbury *et al*. 2019) is concerned with an overall reduction in the use of physical restraint on inpatient wards, but a qualitative analysis of staff perceptions on the training demonstrated a nuanced and enhanced understanding for and compassion towards the person that self‐harms, as well as acknowledgement of the further damage that coercive practice might generate. Our results solely about differential use of these techniques dependent on the behaviour they are intended to manage provide evidence that it is justified to call for minimizing or even eliminating their use specifically for self‐harm management. A move in this direction would also be in keeping with the ideology of trauma‐informed care (TIC). Muskett (2014) describes an increasing awareness and advance towards TIC in mental health inpatient settings, noting that the reduction in seclusion and restraint was both a goal and an outcome. Furthermore, Sweeney and Taggart (2018) note the risk of iatrogenic harm and re‐traumatization when coercive practices reopen the psychological wounds that cause a person to be under nursing care.

Ratings for use of coercive measures for disturbed behaviour did not fall on the disapproving (*M* > 3) side of the scale for any intervention, whereas for use for self‐cutting both physical restraint and forced IM medication were well above that threshold and seclusion almost at it (*M* = 2.97) with close and intermittent observations both close behind. This provides further evidence that study of attitudes to containment measures as a construct needs additional nuancing to account for differences dependent on the reasons for its use. While these attitudes have achieved a certain amount of research attention (Bowers *et al*. 2004, 2007; Whittington *et al*. 2009), they have not been extensively investigated in terms of their relationship with other constructs or with actual practice behaviour (Dickens *et al*. 2022). Partly as a result of this, there is also some room for doubt about the meaning of attitudinal ratings. For example, we do not know whether approval of a particular coercive technique, for example seclusion, is evidence of an over willingness to use it. If so, then presumably we should be aiming to achieve lower levels of approval. However, the current findings, through their demonstration of technique‐oriented attitudinal differentials, suggest that in the case of coercive techniques for self‐harm, specifically restraint, seclusion and forced IM, their use is seriously questionable because they are not even supported in absolute terms. Our previous study (Hosie & Dickens 2018) demonstrated that they were even less well tolerated by the service users at whom they are targeted.

Disapproval of the use of coercive measures for self‐cutting management was greater among those respondents who had not previously used those measures for that purpose. Given the cross‐sectional design of the current study, it is not possible to confidently determine whether this reflects a desensitizing effect whereby once a coercive technique has been used by an individual for self‐cutting management then, for that individual, it becomes more acceptable to do it again. The most obvious alternative is that those who already approve of coercive techniques most from the outset are simply more prepared to use them for any purpose. The phenomenon of professional legitimacy should also be considered at this juncture. McKeown *et al*. (2019) note that mental health services are exceptional in their use of legally sanctioned coercive practice, especially in response to, inter alia, self‐harm. The authors argue that the advent of language about recovery and co‐operation, while commendable, can act as camouflage for disagreeable responses including the use of coercive practices within inpatient settings. Meanwhile, mental health nurses are able to legitimate their coercive response to self‐harm under the banners of safety, necessity and the unalterable last resort. Although self‐rationalized however, it is encouraging that McKeown *et al*. (2020) record both nurses' insight into this process and demonstration of reluctance and doubt. The current study suggests that differences at the individual nurse level play an important role in the process of how coercive practices are legitimized. Further exploration of this phenomenon is warranted and will need to employ longitudinal designs in order to better understand causality. Despite the limitations of the cross‐sectional design here, the linear regression analysis employed suggests that it may be the first alternative. In the case of every coercive intervention examined, the status of the respondent as someone who has previously used techniques for managing self‐cutting was associated with lower disapproval of the use of that technique for managing self‐cutting. The effect from having used the technique for more disturbed behaviour in general usually had no effect on disapproval of the technique for self‐cutting management. Of particular interest, on two occasions when prior use of coercive techniques (physical restraint, seclusion) for disturbed behaviour in general were related to self‐cutting management attitudes, there was an opposite effect, that is the level of disapproval was greater.

All this suggests that when designing staff education about coercive techniques, we need not to only think about the techniques themselves but the purpose for which they are being used. Attitudes to coercive containment techniques are not without nuance and our study demonstrates that they seem to be flexible dependent on what they are being used for. It is of concern that nurses seem to grow used to using these techniques when our previous research shows there is a disparity between them and service users on their acceptability (Hosie & Dickens 2018).

Previous research suggests that attitudes of professionals to self‐harm and to people who harm themselves can be highly polarized (e.g., Patterson *et al*. 2007a, 2007b) including considerable negativity including among mental health nurses. This raises questions about their care of this group. Current management strategies and guidance for self‐harm promote a person‐centred response, but are somewhat vague. For short‐term management, the National Institute for Health and Care Excellence (NICE 2004, 2013) emphasize the importance of risk assessments and mental health triage, a compassionate and respectful response from healthcare practitioners, and a focus on a smooth transition between the various relevant services. Longer term, NICE (2011) suggest reliance on the overall principles of good mental health nursing care; supportive and collaborative relationships, maintaining confidentiality, ongoing assessment of risk, and family involvement where appropriate. Mindful of the perceived difficulties of nursing this behaviour, NICE (2011) also recommends up‐to‐date training and appropriate supervision access. Few specifics are mentioned, beyond access to appropriate psychological interventions and the potential use of harm reduction as a possible alternative approach in the short term. Harm reduction is described as using ‘less destructive’ methods were possible, but no explicit advice is provided.

While ambivalence is prevalent in nurses' mindset towards the harm minimization approach for self‐cutting, methods such as provision of razors or staff remaining present during a cutting event were demonstrably preferable (as evidenced in both nursing staff and service users) to the more commonly used coercive measures of physical restraint and forced administration of IM medication (Hosie & Dickens 2018). In addition, James *et al*. (2017) interviewed mental health professionals, including mental health nurses, on their use of and attitudes towards harm reduction techniques. Those with direct clinical experience remarked that the approach was beneficial to the service users. Participants with no experience of harm reduction methods were understandably concerned with the associated level of risk and the moral and ethical implications. We suggest that it is time for boldness in the approach to the use of harm minimization techniques and one starting point for this is the phasing out of highly coercive containment measures for self‐cutting management.

The study was limited in terms of the sample size and was conducted with an opportunity sample. Replication with a larger sample would be preferable including with non‐UK based mental health nurses. The cross‐sectional design of the study means that we could not make strong, confident claims about causal mechanisms or about whether attitudes might be amenable to change through educational interventions. The generalizability of the findings is limited to self‐cutting specifically since we developed an attitudes scale specifically focused on use of containment measures for this specific behaviour given that we are not aware of any other self‐harm methods that might be amenable to a wide range of harm reduction techniques.

## CONCLUSION

Self‐harm is prevalent in mental health services; many nurses have used coercive techniques to manage self‐cutting in particular, but use of these methods is unpopular with those who have been subject to them for self‐cutting management. The current study shows that mental health nurses' attitudes to these methods has a distinct profile compared with the same methods used for disturbed behaviour in general.

## Relevance for Clinical Practice

The current study suggests that reducing or even eliminating the use of coercive techniques for self‐harm will require attitudes that support its use to be challenged; concurrently, less coercive techniques should be promoted to encourage a more patient‐centred approach. Highly restrictive coercive practices like seclusion, physical restraint, and forced IM medication for managing self‐harm should be discouraged or even banned in all but the most imminent potentially lethal or damaging cases. Concurrently, nurses need to be equipped with the skills and tools to offer more supportive, therapeutic interventions. In our view, harm reduction techniques offer one such alternative.

## Funding

This work was supported by NHS Tayside who funded author LH's Masters By Research Degree. The funder played no role in the conception, design, data analysis, manuscript preparation, or in the decision to publish.
